# Sensory Overresponsivity and Symptoms Across the Obsessive-Compulsive Spectrum: Web-Based Longitudinal Observational Study

**DOI:** 10.2196/37847

**Published:** 2023-04-13

**Authors:** Beatriz Moreno-Amador, Matti Cervin, Agustin Ernesto Martínez-González, Jose A Piqueras, OCD-Spectrum Spain Research Group

**Affiliations:** 1 Area of Personality, Assessment and Psychological Treatment, Department of Health Psychology Universidad Miguel Hernández de Elche Elche Spain; 2 Department of Clinical Sciences Lund Lund University Lund Sweden; 3 Child and Adolescent Psychiatry, Faculty of Medicine Lund University Lund Sweden; 4 Department of Developmental Psychology and Didactics Universidad de Alicante Alicante Spain; 5 See Acknowledgements

**Keywords:** sensory symptoms, sensory overresponsivity, obsessive-compulsive, hair-pulling, skin-picking, hoarding, body dysmorphic, adolescents, adults

## Abstract

**Background:**

Sensory overresponsivity (SOR) has emerged as a potential endophenotype in obsessive-compulsive disorder (OCD), but few studies have examined SOR in relation to the major symptom dimensions of OCD and to symptoms across the full obsessive-compulsive (OC) symptom spectrum.

**Objective:**

This study had 2 main objectives. First, we examined the psychometric properties of the SOR Scales in a community-based sample of Spanish adolescents and adults. Second, we identified how SOR difficulties are related to symptoms across the full OC spectrum (eg, OC, body dysmorphic, hoarding, skin-picking, and hair-pulling symptoms), including the heterogeneity of OC symptoms.

**Methods:**

We translated the SOR Scales into Spanish—a measure that assesses SOR across the 5 sensory modalities—and created a web-based version of the measure. A sample of 1454 adolescents and adults (mean age 23.84, SD 8.46 years) participated in the study, and 388 (26.69%) participants completed the survey twice (approximately 8 months apart). The survey also contained a web-based measure that assesses symptoms across the full OC spectrum: harm and checking, taboo obsessions, contamination or cleaning, symmetry and ordering, body dysmorphic, hoarding, hair-pulling, and skin-picking symptoms.

**Results:**

The psychometric properties of the SOR Scales were excellent, and the test-retest reliability was adequate. All types of SOR were related to all major symptom dimensions of OCD and to all OC spectrum symptoms.

**Conclusions:**

SOR across the sensory modalities can be validly assessed using a web-based measure. SOR emerged as a pure transdiagnostic phenomenon in relation to symptoms across the OC spectrum, with no specific sensory modality being more strongly related to OC symptoms. SOR can shed much needed light on basic mechanisms that are important for the onset and maintenance of OC spectrum symptoms, and this study shows that large-scale web-based studies can aid in this endeavor. Future studies should examine whether SOR precedes or emerges alongside OC symptoms.

## Introduction

### Background

To survive and thrive, humans need to perceive, process, and react to cues in the environment, which can be processed through 5 senses: smell, sight, hearing, touch, and taste. *Sensory integration* (SI), first described by Ayres [1] in 1972, includes the ability to detect, process, and use sensory information to plan and organize behavior [2]. Although all humans are engaged in SI, alterations or disturbances in SI can cause difficulties in sensory processing. To describe difficulties with sensory processing, Miller et al [3] have defined sensory modulation disorders as difficulties in regulating and organizing the type and intensity of behavioral responses to sensory inputs to match environmental demands. Difficulties are organized into three patterns: (1) sensory hyperreactivity or sensory overresponsivity (SOR), (2) sensory hyporeactivity or sensory underresponsivity, and (3) sensation seeking. SOR has been described as a disproportionately intense, prolonged, or intensified reaction to intense distress by sensory stimulation in tactile, sight, auditory, tasting, and smelling sensations, leading to functional impairment [4]. Substantial difficulties with SOR was found in 5% to 21% of school-age children [5,6].

Restricted and repetitive behaviors (RRBs) are behaviors, activities, or interests that occur regularly and interfere with daily performance (eg, stereotypies, restricted interests, and compulsions) [7]. RRBs have emerged as transdiagnostic phenomena in relation to different behavioral, neurological, and neurodevelopmental disorders, such as autism spectrum disorder (ASD), obsessive-compulsive disorder (OCD), and Tourette syndrome, among others [7,8]. Sensory phenomena are perceptions, sensations, or impulses that precede repetitive behaviors [9,10], and studies support a relationship between RRBs and sensory reactivity [11]. Sensory symptoms are often predictive and determining factors in RRBs [12], which tend to develop with some similarity across ASD and OCD [12,13].

Sensory phenomena are more frequently found in patients with a combination of OCD and Tourette syndrome than in patients with OCD only [14], in individuals with early onset OCD [15], and in patients with OCD and chronic tic disorders and a family history of tic disorders [16]. Sensory phenomena are often described as a need to feel *good* internally or a need to calm a generalized internal tension [17], which are similar to the symptoms of hair-pulling disorder (HPD), characterized by a strong urge to pull the hair and the posterior reduction of anxiety or tension [18]. Similarly, sensory symptoms appear in mental disorders characterized by RRBs, such as ASD [19], OCD [20], HPD [21], and attention-deficit/hyperactivity disorder [22]. Specifically, 45% to 95% of individuals with ASD [23,24] and 65% of adults with OCD experience sensory phenomena before ritualization [16], and approximately 15% of individuals with HPD have severe levels of SOR [21].

Sensory reactivity and its links to OCD have been studied in both clinical and nonclinical populations. In a nonclinical adult Israeli sample, a strong association between symptoms of OCD and SOR emerged [25]. Similarly, Taylor et al [26] found 2 classes of individuals in a large nonclinical adult and predominantly US sample: the first class was intolerant of auditory and tactile stimuli, and the second and large class was relatively undisturbed by sensory stimuli. Individuals with sensory intolerance had substantially high scores on obsessive-compulsive (OC) symptoms even after controlling for general psychopathology.

A term closely related to sensory phenomena in OCD is incompleteness. Incompleteness has been shown to be as important for OCD as fear and anxiety, especially in relation to symmetry-related symptoms [27-29]. Accordingly, sensory precursors of OCD symptoms have been suggested to represent a potential marker of putative OCD subtypes [15,30] and may potentially aid in understanding the neuropathology of OCD [16]. Given that many individuals with OCD have an intolerance of *not just right experiences* (NJREs) [31] or imperfect, which is similar to the triggers for many *focused* pulling episodes of HPD, Falkenstein et al [21] proposed that perfectionism, NJREs or incompleteness, and SOR unites the OC spectrum disorders. Lee et al [32] compared adults with OCD with community controls for OC symptoms, perfectionism, and sensory phenomena. Sensory phenomena were more frequent in patients with OCD (68%) than in controls (35%), and many dimensions of perfectionism and sensory phenomena were related. Summers et al [33] examined NJREs in the context of visual, tactile, and auditory modalities in an undergraduate sample. During tasks designed to invoke NJREs, these experiences were associated with perfectionism and experiences across the sensory modalities. Previous studies do not provide information about how SOR is related to different types of OC symptoms, which are highly heterogenous and fall along 3 major symptom dimensions: contamination and cleaning, harm and checking, and symmetry and ordering [34]. This is unfortunate, as studies have shown that the emotional underpinnings of these dimensions are partially distinct with fear mapping onto harm and checking, disgust onto contamination and cleaning, and incompleteness onto symmetry and ordering [28], and it may be that SOR is relevant only for some types of OCD.

The first study that examined SOR in a clinical sample of youth with OCD (aged 3-17 years) found that 32.5% experienced tactile hypersensitivity, 20.3% experienced visual or auditory hypersensitivity, and 20.5% experienced gustatory or olfactory hypersensitivity and that SOR was more common in young children [35]. In the same study, it was found that SOR was most strongly associated with compulsions (vs obsessions) and that sensory phenomena were related to the severity of compulsions, overall OCD burden, and impaired quality of life. A recent study showed that SOR was ubiquitous in youth with OCD; not explained by co-occurring ASD or attention-deficit/hyperactivity disorder; and linked to all OCD dimensions, depression, and anxiety; however, SOR was equally common among youth with anxiety disorders [36]. A recent study that included adults found no differences in SOR among those with chronic tic disorders, OCD, and OCD along with chronic tic disorders, and all 3 groups had more difficulties with SOR than healthy individuals, pointing toward transdiagnostic features of SOR [37].

Previous studies largely miss information about how SOR is related to the broad OC symptom spectrum, which in addition to OCD, includes hair-pulling, skin-picking, hoarding, and body dysmorphic symptoms. Individuals with body-focused repetitive behaviors (BFRBs), such as HPD and skin-picking disorder, have elevated perceptions of somatic activity (eg, itchy scalp). A study examined somatic activity in BFRBs and found that HPD and skin-picking disorder were associated with great awareness of somatic sensations [38]. Another study showed that those with BFRBs had increased sensory sensitivity and sensory avoidance, but no differences in sensation seeking, compared with those with subclinical BFRBs and those without BFRBs [39]. A recent study with an adult population showed that high levels of SOR were related to high levels of *focused* types of HPD, perfectionism, and proxy-pulling urges toward other people, suggesting that SOR may be related to maladaptive emotion regulation processes in HPD. Similarly, the study found high levels of BFRBs in those with great awareness of bodily sensations [21]. Thus, it appears that SOR may be a common mechanism running through symptoms across the OC symptom spectrum, but few studies have been conducted and no study has analyzed SOR in relation to the full OC spectrum.

Several scales are currently used to analyze sensory symptoms, for example, the *Short Sensory Profile 2* [40]; *Sensory Experiences Questionnaire Version 3.0* [41]; *Glasgow Sensory Questionnaire* [42]; *Sensory Over-Responsivity Inventory* [43]; *Sensory Sensitivity Scales* [44]; *Sensory Perception Quotient—short version* [45]; and *SOR Scales* that include modules assessing SOR related to smell, sight, taste, hearing, and touch [21]. No self-report scales for SOR have been validated in the Spanish language. A good self-report candidate is the SOR Scales as they assess SOR across the 5 sensory modalities, which may be important in the assessment of individuals with neurodevelopmental disorders, mental disorders, brain damage, and other types of disorders.

### Objectives

This study had 2 main objectives. First, we examined the psychometric properties of the SOR Scales in a community-based sample of Spanish adolescents and adults. Second, we identified how SOR difficulties are related to symptoms across the full OC spectrum (eg, OC, body dysmorphic, hoarding, skin-picking, and hair-pulling symptoms), including the heterogeneity of OC symptoms.

## Methods

### Participants and Procedure

The data analyzed in this study were drawn from a prospective cohort study with 3 evaluations over a period of 18 months. In this study, data from the first (time 0 [T0]) and second (time 1 [T1]) data collections were analyzed (the third data collection is ongoing). The sample consisted of 1454 (T0) and 388 (T1; 388/1454, 26.69% of the T0 sample and 388/1162, 33.39% of the people who received the T1 reminder) Spanish adolescents and adults aged 14 to 64 years (T0: mean age 23.84, SD 8.46 years and T1: mean age 26.22, SD 7.51 years) who completed all measures of the study. Participants were recruited through web-based advertisement, specifically through organizations associated with mental health, especially those pertaining to OCD and BFRBs, and through advertisements in secondary education centers and universities. This strategy was used to oversample individuals with high levels of symptoms across the OC spectrum, which is consistent with the Research Domain Criteria recommendation for linking mechanisms and psychopathology dimensions [46]. The participants did not provide any information regarding how they had encountered the study.

This study was funded by the Alicia Koplowitz Foundation, a nonprofit organization with the objective to promote training and research in child and adolescent psychiatry. The first phase of the study was conducted between October 2020 and October 2021 and the second phase between June 2021 and December 2021, with a time lapse of approximately 8 (SD 1.25) months between the first and second assessments. Differences in duration between T0 and T1 emerged because several participants (990/1162, 85.19%) did not complete the follow-up survey directly when they received the email invitation. If a participant did not respond, reminder emails were sent. This led to several participants (216/1162, 18.59%) completing the survey after several reminders. The second reminder was sent approximately 7 months after the completion of the first evaluation and only to the people who completed the first assessment between October 2020 and March 2021.

Participants completed the study measures via the web, using the web-based survey tool, LimeSurvey (LimeSurvey GmbH), for both assessments. At the beginning of each questionnaire, every participant had to enter a unique code generated by LimeSurvey and an email for future participation in the study. All codes and emails were analyzed to ensure that no participant responded multiple times. If multiple responses were present, only 1 survey was included, and we prioritized the most complete survey or the first survey if both surveys were completed. Participation was voluntary, and participants had the chance to win a gift card worth €100 (US $108.07).

Table 1 presents the sociodemographic information of T0 and T1 samples.

**Table 1:** Sociodemographic characteristics of the sample.

Variables			Time-0 sample		Time-1 sample
**Age (years; time 0: n=1454; time 1: n=388), mean (SD)**			23.84 (8.46)		26.22 (7.51)
	14-18, n (%)	469 (32.26)		20 (5.2)	
	19-25, n (%)	580 (39.89)		208 (53.6)	
	26-30, n (%)	194 (13.34)		87 (22.4)	
	>30, n (%)	211 (14.51)		73 (18.8)	
**Sex (time 0: n=1454; time 1: n=388), n (%)**					
	Female	1041 (71.59)		305 (78.6)	
	Male	404 (27.79)		81 (20.9)	
	Other	1 (0.07)		0 (0)	
	Not want to answer	8 (0.55)		2 (0.5)	
**Gender (time 0: n=1438; time 1: n=388), n (%)**					
	Women	1028 (71.48)		301 (77.6)	
	Men	397 (27.61)		79 (20.4)	
	Other	3 (0.21)		6 (1.5)	
	Not want to answer	10 (0.69)		2 (0.5)	
**Country or region of birth (time 0: n=1454; time 1: n=388), n (%)**					
	Spain	1341 (92.23)		383 (98.7)	
	Rest of Europe	32 (2.20)		5 (1.3)	
	America	66 (4.54)		0 (0)	
	Africa	10 (0.69)		0 (0)	
	Asia	5 (0.34)		0 (0)	
**Country or region of residence (time 0: n=1453; time 1: n=388), n (%)**					
	Spain	1438 (98.96)		385 (99.2)	
	Rest of Europe	14 (0.96)		3 (0.8)	
	Asia	1 (0.07)		0 (0)	
**Spanish participants (time 0: n=1454; time 1: n=388), n (%)**					
	Origin and residence in Spain	1325 (91.13)		360 (92.8)	
	Origin or residence in Spain	129 (8.87)		28 (7.2)	
**Education level (time 0: n=1214; time 1: n=353), n (%)**					
	Secondary	448 (36.90)		109 (30.9)	
	Superior	766 (63.09)		244 (69.1)	
**Presence of psychological problems (time 0: n=1454; time 1: n=388), n (%)**					
	Body-focused repetitive behaviors	135 (9.28)		51 (13.1)	
	Obsessions	147 (10.11)		49 (12.6)	
	Compulsions	86 (5.91)		27 (6.9)	

### Ethics Approval

The study was approved by the Universidad Miguel Hernández Project Evaluation Committee (DPS.JPR.03.20).

### Measures

#### The SOR Scales

The SOR Scales [21] is a new instrument accessible to the community. This self-reported measure assesses hypersensitivity across the 5 senses: smell, sight, taste, hearing, and touch. The SOR Scales was adapted from a measure used with a general community sample in a survey study [26]. Each of its domains contains 4 questions rated on a scale from 0 to 4, with an overall score ranging from 0 to 80. The total scores for each subscale range from 0 to 16, with high scores indicating great SOR. The scale items pertain to distress, avoidance, control, and social or work-related impairment. The psychometric properties of the measure, including model or data fit and internal consistency of each domain, were analyzed in this study.

#### The OC and Related Disorders Dimensional Scales–Expanded Version

To measure symptoms across the major symptom dimensions of OCD and across the OC spectrum, we took advantage of recent advances in the measurement of OCD and related disorders by using an updated version of the Obsessive-Compulsive Dimensional Scales [47] that assesses body dysmorphic, hoarding, skin-picking, and hair-pulling symptoms and the 4 major symptom dimensions of OCD (contamination and cleaning, taboo obsessions, harm and checking, and symmetry and ordering) [48]. Thus, the scale yields a measurement of 8 OC symptom dimensions, and each dimension is assessed using 5 items: time, control, distress, avoidance, and interference. The measure shows excellent psychometric properties, which are reported in detail elsewhere [48]. The internal consistency for each scale in the present sample was good to excellent: OCD–harm and checking (Cronbach α=.89), OCD–taboo obsessions (Cronbach α=.92), OCD–symmetry and ordering (Cronbach α=.91), OCD–contamination and cleaning (Cronbach α=.95), body dysmorphic (Cronbach α=.95), hoarding (Cronbach α=.84), hair pulling (Cronbach α=.95), and skin picking (Cronbach α=.90).

### Data Analyses

We examined the psychometric properties of the SOR Scales using a 3-stage approach. First, we fitted the proposed 5-factor structure of the scale (the 5 sensory modalities) to the data collected at the first assessment, using confirmatory factor analysis. Factor analysis was conducted using R Studio (version 2021.09.0) using the R library, *lavaan*. Diagonally weighted least-squares estimation was used because of ordinal response items. Fit indexes (indicating to which degree a proposed factor structure fits data) were examined, and scaled versions of the confirmatory fit index (CFI), root mean square error of approximation (RMSEA), standardized root mean square residual (SRMR), and Tucker-Lewis fit index (TLI) were computed and examined. No single index was considered more important than the others; instead, a global evaluation was conducted. Adequate model fit was considered to be indicated by CFI and TLI values >0.95, a RMSEA value <0.06, and an SRMR value of 0.08. The model or data fit of the proposed 5-factor model was compared with the model or data fit of a unidimensional model, in which all SOR items loaded onto a single SOR factor, and with a 2-factor model, in which distress or avoidance loaded onto one factor and the 2 interference (social, work, or home) items loaded onto another factor.

After model or data fit evaluation, we examined the internal consistency of the items of each SOR subscale. Both Cronbach α and McDonald Ω were calculated. Finally, we examined test-retest reliability by correlating scores at T0 with scores at T1 for the subsample that completed the measure on both occasions (388/1454, 26.69%); Pearson correlation coefficients were estimated.

To examine the associations between SOR factors and OCD spectrum factors, we used data from the first assessment (because of the large sample size) and modeled the data using structural equation modeling. The proposed SOR model was used as the measurement model for SOR, and the 8 OC symptom dimensions from the Obsessive-Compulsive and Related Disorders Dimensional Scales–Expanded Version were used as the measurement model for the OC spectrum factors. At the structural level, we estimated covariance (ie, correlation) parameters between SOR and OCD spectrum factors, which allowed us to examine the degree to which the different SOR factors were related to the different OC spectrum factors. Furthermore, to examine the unique associations between SOR and OC spectrum symptoms, we used regression and used the different OC spectrum symptoms as the dependent variables and the SOR factors as independent variables, while accounting for age and sex. The OC spectrum symptom data were 0 inflated, that is, many participants reported the minimum score. The 0 inflation ranged from 19.25% (280/1454) in symmetry and ordering to 77.65% (1129/1454) in hair pulling. The data were also overdispersed, with negative binomial distribution and variance that was larger than the mean. To model these data, we used 0-inflated negative binomial regression, which estimates the association between independent variables and a dependent variable that contains nonnegative integers with a binomial distribution. The model yields two outputs: (1) the probability of having no symptoms (expressed as odds ratios) and (2) increasing scores on the dependent variable (expressed as incidence rate ratio). The 0-inflated negative binomial regression models were also used to examine whether SOR predicted OC spectrum scores at the second assessment, while accounting for the first assessment of OC spectrum scores, age, and sex. As we used several statistical models, Cronbach α level of .01 was used as an indicator of statistical significance.

## Results

### Psychometric Properties of the SOR Scales

Detailed model data fit indexes for all models are presented in Table 2. The proposed 5-factor structure of the SOR Scales had excellent model or data fit according to all fit indexes, except the RMSEA index, which was in the good range. The model or data fit of the proposed factor structure was clearly superior compared with the 1-factor and 2-factor models, of which neither showed adequate fit. The 5-factor model fitted equally well when we used data from the second assessment. In the 5-factor model, all items loaded statistically significantly onto their proposed factor (*P*<.001), with no standardized loading being below .80 and the highest being .95. All standardized factor loadings are presented in Table S1 in Multimedia Appendix 1.

In the 5-factor model, we let the 5 latent SOR factors to correlate freely, and these correlations ranged from *r*=0.41 to *r*=0.54. Thus, relations among the factors were all in a similar range. Accordingly, an exploratory factor analysis using principal axis factoring and promax rotation suggested a 1-factor solution for the SOR factors. Thus, we tested whether a high-order model in which a high-order SOR factor explained the pattern of covariation among the 5 SOR factors had adequate fit. Fit indexes for the constrained high-order model are shown at the bottom of Table 2 (fitted to both T0 and T1 data). Results showed that fit indexes were better for the high-order model and clearly improved for indexes that accounted for parsimony (ie, RMSEA, CFI, and TLI). We interpreted this as evidence that the pattern of covariation among the SOR factors could be adequately accounted for by a single overarching SOR factor.

The internal consistency for the items of each SOR factor (ie, the 5 sensory factors) was excellent: hearing (Cronbach α=.89; McDonald Ω=0.90), touch (Cronbach α=.86; McDonald Ω=0.92), sight (Cronbach α=.90; McDonald Ω=0.95), smell (Cronbach α=.91; McDonald Ω=0.96), and taste (Cronbach α=.86; McDonald Ω=0.94). Test-retest reliability was adequate for the 5 factors (touch: *r*=0.60; hearing: *r*=0.64; smell: *r*=0.64; sight: *r*=0.57; and taste: *r*=0.58) and good for the full scale (*r*=0.75). Table 3 shows the means and SDs for the 5 scales or factors of the SOR Scales for the full sample at T0 and for the sample that completed the scale at the first and second assessments.

We proceeded to compare scores within the different SOR factors. Paired samples *t* tests (2 tailed) showed that the mean scores on all scales differed statistically significantly from each other (all *P*<.05). The highest mean score was found for hearing, followed by touch, smell, sight, and taste. Effect sizes ranged from Cohen *d*=0.06 (for the difference between smell and sight) to Cohen *d*=0.54 (for the difference between hearing and taste). Detailed results are provided in Table S2 in Multimedia Appendix 1.

**Table 2:** Fit indexes for the proposed 5-factor structure of the Sensory Over-Responsivity Scales in comparison with 1-factor and 2-factor structures. The model fitted with T0^a^ data used data from 1454 individuals. The model fitted with T1^b^ data used data from 485 individuals.

	Chi-square (*df*)	*P* value^c^	CFI^d^	TLI^e^	RMSEA^f^	SRMR^g^
Proposed 5-factor structure (T0)	1127.2 (160)	<.001	0.979	0.975	0.064	0.048
Unidimensional factor structure (T0)	9891.4 (170)	<.001	0.792	0.768	0.198	0.204
2-factor structure (distress or avoidance and impairment; T0)	9839.4 (179)	<.001	0.793	0.767	0.198	0.203
Proposed 5-factor structure (T1)	467.6 (160)	<.001	0.984	0.981	0.063	0.053
High-order 5-factor model (T0)	948.3 (165)	<.001	0.983	0.981	0.057	0.056
High-order 5-factor model (T1)	388.3 (165)	<.001	0.988	0.987	0.053	0.056

^a^T0: time 0.

^b^T1: time 1.

^c^The *t* test was 2 tailed.

^d^CFI: confirmatory fit index.

^e^TLI: Tucker-Lewis fit index.

^f^RMSEA: root mean square error of approximation.

^g^SRMR: standardized root mean square residual.

**Table 3:** Means and SDs of the total score and each dimensional score of the Sensory Over-Responsivity Scales at T0^a^ and T1^b^.

Sensory Over-Responsivity Scales score, range	T0 full sample (n=1454), mean (SD)	T1 full sample (n=388), mean (SD)
0-16	2.98 (2.97)	3.15 (2.82)
0-16	4.16 (3.71)	4.76 (3.57)
0-16	2.47 (3.20)	2.56 (3.09)
0-16	2.28 (2.97)	2.42 (2.89)
0-16	2.03 (2.79)	2.08 (2.60)
0-80	13.91 (11.18)	14.97 (10.59)

^a^T0: time 0.

^b^T1: time 1.

### Associations Between SOR and Symptoms of the OCD Spectrum

**Figure 1:**
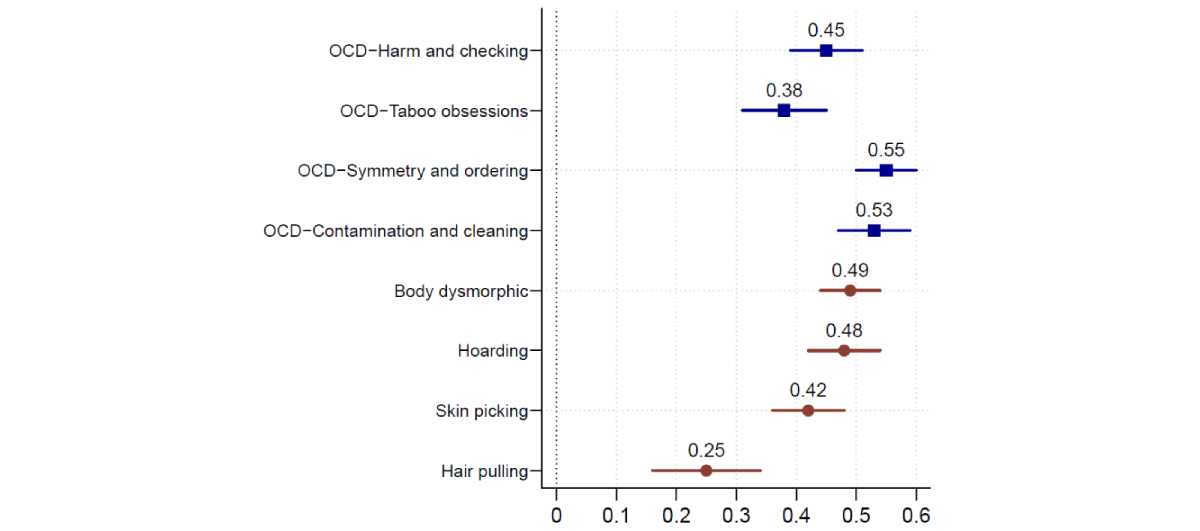
Associations in the form of standardized covariance coefficients and their 95% CIs between the broad sensory overresponsivity factor and the obsessive-compulsive disorder (OCD) spectrum factors.

We fitted a model in which SOR and OCD spectrum factors were all included in the measurement model. We constrained the 5 SOR factors to load onto the high-order SOR factor, which was allowed to correlate freely with the OC spectrum factors. The model or data fit was good (CFI=0.987; TLI=0.986; RMSEA=0.032; and SRMR=0.046), and the associations (correlations) between the broad SOR factor and each of the OC spectrum factors are presented in Figure 1. The broad SOR factor correlated statistically significantly with all OC spectrum factors; the association with symmetry or ordering was the largest, and the association with hair pulling was the smallest.

To estimate the unique associations between the total SOR score and OC spectrum factors, we used 0-inflated negative binomial regression. Detailed results are presented in Tables S3 and S4 in Multimedia Appendix 1. First, we examined the probability of reporting no symptoms versus any symptoms. The total SOR score was a statistically significant predictor of all OC spectrum symptoms (*P*<.001), and all models indicated that higher SOR was associated with low probability of reporting no symptoms, with the strongest association being found in relation to symmetry and ordering. Age and sex were included as covariates in all models, and old age was associated with high probability of reporting no symmetry and ordering, body dysmorphic, and skin-picking symptoms. Being male was significantly associated with low probability of reporting no taboo obsession symptoms (*P*<.001), and being female was associated with low probability of reporting no body dysmorphic symptoms (*P*<.001). Similarly, high SOR scores were significantly associated with high scores on all OC spectrum scales (*P*<.001) except hair-pulling symptoms. Older age was significantly associated with higher scores on all OC spectrum factors (*P*<.01), except body dysmorphic, hoarding, and skin-picking symptoms. Being male was significantly associated with low scores on contamination and cleaning (*P*<.01), body dysmorphic (*P*<.001), and skin-picking symptoms (*P*<.001).

When predicting OC spectrum symptom factors at T1 using the total SOR score at T0 while accounting for age, sex, and T0 score on the same OC spectrum factor, SOR significantly predicted low probability of reporting no symptoms at T1 for taboo obsessions (*P*<.01) and hoarding (*P*<.01). Scores on the same OC spectrum symptom factor significantly predicted reporting symptoms for all OC spectrum symptoms (*P*<.001), except symmetry and ordering. When predicting increasing scores on the OC spectrum factors at T1, SOR was a significant predictor only for harm and checking (*P*<.01). Again, the severity of the same OC spectrum factor was the clearest predictor and significantly predicted severity for all OC spectrum factors at T1 (all *P*<.001). Refer to Tables S3 and S4 in Multimedia Appendix 1 for detailed results.

## Discussion

### Principal Findings

In this study, we showed that a Spanish web-based version of the SOR Scales has strong psychometric properties and a sound 5-factor structure consistent with the proposed theoretical structure of the measure [21]. This is the first empirical evidence supporting the factor structure of the SOR Scales, and our findings show that SOR can be measured in a valid way using a web-based measure using this scale. Web-based surveys of SOR are an effective way to increase sample sizes in future studies, and the SOR Scales can aid in such studies. However, the measure may also serve the purpose of a brief screening measure of sensory difficulties across the 5 senses in clinical practice and clinical research. Researchers can select whether to analyze the broad SOR dimension or the 5 subdimensions depending on their research questions.

Regarding links between SOR and OC spectrum symptoms, the overall results of this study suggest that SOR is related to all the major OC spectrum symptoms, with no clear indications that it is more strongly associated with certain symptoms. When examining whether SOR predicted later OC spectrum symptoms, weak evidence emerged for SOR as a significant predictor. However, the study only included 2 assessments conducted 8 months apart, and it is highly likely that causal or reciprocal relations between SOR and OC spectrum symptoms may only be uncovered using more advanced temporal designs, for example, by following individuals from childhood to adulthood.

Previous studies have highlighted the importance of sensory reactivity in both clinical and nonclinical populations [15,25,26]. The development of self-reported instruments such as the SOR Scales allows advance in the study of sensory reactivity in humans. To the best of our knowledge, this is the first study to analyze SOR in relation to the full OC spectrum, including the heterogeneity of OCD, with previous studies being characterized by analyzing SOR in relation to 1 symptom dimension at the time, for example, OCD and HPD [21,25]. Our results are consistent with those of other studies in which sensory experiences have been linked to OCD, especially in relation to symmetry-related symptoms [27-29], and with a study by Lewin et al [35], in which SOR was more strongly associated with compulsions than obsessions in children with OCD. Interestingly, the strongest association between SOR and OC spectrum dimensions that emerged in this study was between SOR and symmetry and ordering.

Previous studies have implicated SOR, particularly in relation to BFRBs (eg, hair-pulling and skin-picking symptoms), and has explored SOR mainly in relation to hair pulling [38,39]. Moreover, hair pulling and skin picking have been suggested to be characterized more by compulsiveness than by obsessiveness, with the latter being more present in OCD, body dysmorphic disorder, and hoarding disorder [49]. Furthermore, there is evidence suggesting that sensory phenomena generate compulsive or ritualistic behaviors [14]. Thus, previous findings suggest that SOR would be most clearly indicated in hair pulling and skin picking. If anything, we found that SOR was less strongly related to these types of symptoms. Thus, the results of this study challenge the notion that SOR is most strongly indicated in OC spectrum difficulties characterized by compulsivity. Rather, our results support that SOR is a transdiagnostic factor linked to all forms of OC spectrum symptoms.

The SOR domain in which most individuals experienced difficulties was hearing, followed by touch, smell, sight, and taste. These results moderately contradict with those of a previous study examining SOR in a pediatric OCD sample, where 32.5% experienced tactile hypersensitivity, 20.3% experienced visual or auditory hypersensitivity, and 20.5% experienced gustatory or olfactory hypersensitivity [35]. Differences may be explained by recruitment (our sample was a diverse sample recruited using a web-based survey) and differences in age and the proportion of men and women (most individuals in our sample were women—1028/1454, 70.7%—whereas the pediatric sample consisted mostly of boys) [50]. Consistent with our results, a nonclinical study of adults indicated that auditory and tactile intolerances showed the highest correlation with OC symptoms [25]. Similarly, studies using magnetoencephalography in youth and adults suggest that individuals with OCD have altered sensory processing of auditory information compared with healthy individuals [51,52]. Although there is still little research on this issue, it can be stated that tactile and acoustic modalities of stimulation are the most investigated according to a recent systematic review focused on sensory phenomena in OCD [53].

### Limitations

Some methodological limitations should be noted. First, an inherent limitation of this study is the exclusive use of self-reports to assess SOR and OC spectrum symptoms, with general limitations of self-report measures applying here, such as social desirability, response bias, or introspective inability of responders. However, a recent study that focused on differentiating between sensory sensitivity and sensory reactivity in relation to repetitive behaviors and restricted interests showed that self-report measures better captured sensory reactivity, whereas behavioral measures were better measures of sensory sensitivity [54]. Future studies on SOR and the OC spectrum should use other methods to generalize our findings (eg, multiple informants) and include social desirability scales or infrequency scales to detect random responses. Second, we were unable to examine the relationships between the SOR Scales and other previously validated questionnaires. However, the only validated test in Spain, the *Short Sensory Profile 2* by Dunn [40], is focused on multiple informants reporting the sensory sensitivity of children aged from 3 to 14 years; therefore, it was not applicable in this study. Third, only approximately one-fourth of the sample (388/1454, 26.69%) completed the second (T1) survey, which may have introduced attrition bias. Fourth, the samples were not fully representative of the Spanish population; therefore, the generalizability of the results is limited. Finally, although we tried to oversample individuals experiencing SOR and OC spectrum symptoms, many scales were 0 inflated. Future studies may include pure clinical samples also.

### Conclusions

In conclusion, SOR can be measured adequately using a web-based version of the SOR Scales, and this study showed that SOR appears to be a transdiagnostic phenomenon related to symptoms across the OC spectrum. As such, SOR holds promise as a potential mechanism that can help to elucidate the complex etiology of OC spectrum disorders. However, future studies need to establish whether difficulties with SOR precede OC spectrum symptoms, arise alongside them, or are a secondary or epiphenomenal consequence. In addition, studies using clinical populations are needed to generalize the present findings to a clinical context, and studies on SOR in relation to variables associated with the gut microbiota–brain axis (eg, gastrointestinal symptoms and pain) may help to further elucidate the nature of SOR across the OC spectrum.

## Appendix

Multimedia Appendix 1 Supplementary tables.

## Abbreviations

ASD: autism spectrum disorder
BFRB: body-focused repetitive behavior
CFI: confirmatory fit index
HPD: hair-pulling disorder
NJRE: not just right experience
OC: obsessive-compulsive
OCD: obsessive-compulsive disorder
RMSEA: root mean square error of approximation
RRB: restricted and repetitive behavior
SI: sensory integration
SOR: sensory overresponsivity
SRMR: standardized root mean square residual
T0: time 0
T1: time 1
TLI: Tucker-Lewis fit index
