# Supplementary material for: Sensory Overresponsivity and Symptoms Across the Obsessive-Compulsive Spectrum: Web-Based Longitudinal Observational Study
Source: J Med Internet Res. 2023 Apr 13;25:e37847. doi: 10.2196/37847 (PMC10141273; doi:10.2196/37847)
Supplement: Multimedia Appendix 1 [file jmir_v25i1e37847_app1.docx]

**Multimedia Appendix 1**

**Supplementary information**

for

Sensory over-responsivity and symptoms across the obsessive-compulsive spectrum:

a web-based study

**Table S1.** Standardized factor loadings for the five-factor structure of the Sensory Over-Responsivity Scales based on confirmatory factor analysis.

|  |  | Standardized factor loading |
| --- | --- | --- |
| **Touch** | |  |
|  | *Item 1. Distress* | 0.80 |
|  | *Item 2. Avoidance* | 0.89 |
|  | *Item 3. Control* | 0.88 |
|  | *Item 4. Interference* | 0.89 |
| **Hearing** | |  |
|  | *Item 1. Distress* | 0.86 |
|  | *Item 2. Avoidance* | 0.91 |
|  | *Item 3. Control* | 0.86 |
|  | *Item 4. Interference* | 0.90 |
| **Smell** | |  |
|  | *Item 1. Distress* | 0.88 |
|  | *Item 2. Avoidance* | 0.95 |
|  | *Item 3. Control* | 0.94 |
|  | *Item 4. Interference* | 0.94 |
| **Sight** | |  |
|  | *Item 1. Distress* | 0.87 |
|  | *Item 2. Avoidance* | 0.93 |
|  | *Item 3. Control* | 0.93 |
|  | *Item 4. Interference* | 0.94 |
| **Taste** | |  |
|  | *Item 1. Distress* | 0.89 |
|  | *Item 2. Avoidance* | 0.93 |
|  | *Item 3. Control* | 0.88 |
|  | *Item 4. Interference* | 0.93 |

**Table S2.** Results from dependent samples t-tests comparing the different factors of the Sensory Over-Responsivity Scales.

|  | Difference | 95% CI for difference | *t* | *p* | *d* |
| --- | --- | --- | --- | --- | --- |
| Touch vs Hearing | -1.176 | -1.364, -.988 | -12.252 | <.001 | -.321 |
| Touch vs Smell | 0.512 | 0.344, 0.680 | 5.978 | <.001 | .157 |
| Touch vs Sight | 0.702 | 0.538, 0.867 | 8.373 | <.001 | .220 |
| Touch vs Taste | 0.956 | 0.798, 1.114 | 11.849 | <.001 | .311 |
| Hearing vs Smell | 1.688 | 1.488, 1.888 | 16.537 | <.001 | .434 |
| Hearing vs Sight | 1.878 | 1.693, 2.064 | 19. 846 | <.001 | .520 |
| Hearing vs Taste | 2.132 | 1.928, 2.336 | 20.533 | <.001 | .538 |
| Smell vs Sight | 0.191 | 0.012, 0.369 | 2.091 | .018 | .055 |
| Smell vs Taste | 0.444 | 0.278, 0.611 | 5.238 | <.001 | .137 |
| Sight vs Taste | 0.254 | 0.076, 0.431 | 2.806 | .003 | .074 |

**Table S3.** Results (odds ratios) from zero-inflated negative binomial regression predicting OC spectrum factors at T0 and T1 (no symptoms versus any symptom) using the total SOR score (scale: 0-80), age, and sex as predictors. When predicting T1 OC spectrum symptoms, T0 OC spectrum severity for that OC spectrum factor was included as an additional covariate.

|  | Harm and checking  T0 | Taboo obsessions T0 | Symmetry and  ordering T0 | Contamination and  cleaning T0 | Body dysmorphic T0 | Hoarding  T0 | Hair-pulling  T0 | Skin-picking  T0 |
| --- | --- | --- | --- | --- | --- | --- | --- | --- |
| Total SOR score T0 | 0.929** | 0.944** | 0.896** | 0.942** | 0.925** | 0.928** | 0.963** | 0.955** |
| Age in years | 1.012^ns^ | 1.013^ns^ | 1.035** | 1.007^ns^ | 1.043** | 1.004^ns^ | 0.986^ns^ | 1.025^**^ |
| Sex (male vs female) | 1.037^ns^ | 0.494** | 1.168^ns^ | 1.100^ns^ | 1.774** | 0.934^ns^ | 1.217^ns^ | 1.349^ns^ |
|  | Harm/checking  T1 | Taboo obsessions T1 | Symmetry/  ordering T1 | Contamination/  cleaning T1 | Body dysmorphic T1 | Hoarding  T1 | Hair-pulling  T1 | Skin-picking  T1 |
| Total SOR score T0 | 0.973^ns^ | 0.963* | 0.913^ns^ | 0.988^ns^ | 0.970^ns^ | 0.942* | 0.988^ns^ | 0.970^ns^ |
| Age in years | 1.033^ns^ | 1.032^ns^ | 1.064^ns^ | 1.047* | 1.026^ns^ | 1.043^ns^ | 0.999^ns^ | 1.040^ns^ |
| Sex (male vs female) | 0.525^ns^ | 0.470^ns^ | 0.378^ns^ | 0.902^ns^ | 0.835^ns^ | 0.940^ns^ | 0.788^ns^ | 1.269^ns^ |
| Score on the same OC factor T0 | 0.780** | 0.735** | 0.513^ns^ | 0.798** | 0.793** | 0.309** | 0.700** | 0.716** |

Notes. ^ns^ Not statistically significant. * p < .01. ** p < .001. OC = Obsessive-Compulsive. SOR = Sensory Over-Responsivity. T0 = Assessment 1. T1 = Assessment 2 (approximately 8 months later).

**Table S4.** Results (incidence rate ratios) from zero-inflated negative binomial regression predicting OC spectrum factors at T0 and T1 (scale: 0-20) using the total SOR score (scale: 0-80), age, and sex as predictors. When predicting T1 OC spectrum symptoms, T0 OC spectrum severity for that OC spectrum factor was included as an additional covariate.

|  | Harm and checking  T0 | Taboo obsessions T0 | Symmetry and  ordering T0 | Contamination and  cleaning T0 | Body dysmorphic T0 | Hoarding  T0 | Hair-pulling  T0 | Skin-picking  T0 |
| --- | --- | --- | --- | --- | --- | --- | --- | --- |
| Total SOR score T0 | 1.015** | 1.014** | 1.024** | 1.022** | 1.017** | 1.019** | 1.001^ns^ | 1.018** |
| Age in years | 1.016** | 1.020* | 1.012* | 1.014** | 0.996^ns^ | 1.009^ns^ | 1.028** | 1.010^ns^ |
| Sex (male vs female) | 0.873^ns^ | 1.096^ns^ | 0.892^ns^ | 0.780* | 0.722** | 0.965^ns^ | 0.703^ns^ | 0.691** |
|  | Harm/checking  T1 | Taboo obsessions T1 | Symmetry/  ordering T1 | Contamination/  cleaning T1 | Body dysmorphic T1 | Hoarding  T1 | Hair-pulling  T1 | Skin-picking  T1 |
| Total SOR score T0 | 1.013* | 1.007^ns^ | 1.001^ns^ | 1.001^ns^ | 1.004^ns^ | 1.001^ns^ | 1.001^ns^ | 1.003^ns^ |
| Age in years | 0.995^ns^ | 1.015^ns^ | 1.000^ns^ | 0.999^ns^ | 1.001^ns^ | 1.001^ns^ | 0.996^ns^ | 0.993^ns^ |
| Sex (male vs female) | 0.781^ns^ | 0.782^ns^ | 0.825^ns^ | 0.952^ns^ | 0.889^ns^ | 0.693^ns^ | 0.827^ns^ | 0.909^ns^ |
| Score on the same OC factor T0 | 1.081** | 1.078** | 1.100** | 1.083** | 1.062** | 1.084** | 1.093** | 1.067** |

Notes. ^ns^ Not statistically significant. * p < .01. ** p < .001. OC = Obsessive-Compulsive. SOR = Sensory Over-Responsivity. T0 = Assessment 1. T1 = Assessment 2 (approximately 8 months later).
